# Pre-Operative Cognitive Functioning and Inflammatory and Neuroendocrine Responses to Cardiac Surgery

**DOI:** 10.1007/s12160-016-9779-7

**Published:** 2016-02-10

**Authors:** Lydia Poole, Amy Ronaldson, Tara Kidd, Elizabeth Leigh, Marjan Jahangiri, Andrew Steptoe

**Affiliations:** Department of Epidemiology and Public Health, University College London, 1-19 Torrington Place, London, WC1E 6BT UK; Department of Cardiac Surgery, St George’s Hospital, University of London, Blackshaw Road, London, SW17 0QT UK

**Keywords:** Cognition, Coronary artery bypass graft surgery, Inflammation, Cortisol

## Abstract

**Background:**

Cognitive functioning is linked to cardiac mortality and morbidity, but the mechanisms underlying this relationship are unclear.

**Purpose:**

To examine the relationship between pre-operative cognitive functioning and post-operative inflammatory and neuroendocrine responses in patients undergoing coronary artery bypass graft (CABG) surgery.

**Methods:**

One-hundred ninety-three outpatients were screened to assess their cognitive function using the Montreal Cognitive Assessment (MoCA) on average 30 days prior to CABG surgery and provided blood samples for the measurement of interleukin (IL)-6 and C-reactive protein (CRP) and saliva samples for the measurement of diurnal cortisol. Participants were followed-up 4–8 days following surgery for the repeat measurement of IL-6 and CRP and 60 days after surgery for the measurement of diurnal salivary cortisol.

**Results:**

Patients with low cognitive function (MoCA < 26) prior to surgery reached higher IL-6 concentrations in the days after surgery (*β* = −0.212, *p* = 0.021) and had greater cortisol output across the day 2 months after surgery (*β* = −0.179, *p* = 0.044).

**Conclusions:**

Low cognitive functioning is associated with a more negative pattern of biological response to surgery, indicative of poorer physical recovery. These pathways may contribute to the links between cognitive function and cardiovascular pathology.

## Introduction

Cardiovascular risk factors are known to be associated with cognitive decline [1] and dementia [2]. Conversely, low levels of cognitive function have long been associated with greater all-cause [3] and cardiac mortalities [4]. In particular, much attention has been paid to cognitive function and coronary artery bypass graft (CABG) surgery, with estimates suggesting that cognitive impairment is present in approximately 35 % of patients prior to surgery [5] and in between 22.5 and 42 % of CABG patients following surgery [6, 7]. The mechanisms linking cognitive impairment and cardiac disease are not clearly understood but are likely to include both inflammatory and neuroendocrine pathways.

Whilst mixed evidence exists, inflammation is thought to be a common causal factor in both coronary heart disease (CHD) [8, 9] and cognitive decline [10]. CABG surgery is associated with an acute inflammatory response. The extent of the inflammatory response is thought largely to reflect the amount of trauma derived from the surgical procedure itself and is associated with a host of clinical outcomes [11, 12]. Likewise, heightened levels of C-reactive protein (CRP) have been associated cross sectionally with mild cognitive impairment [13] and there is some evidence for a longitudinal effect, with both CRP and interleukin (IL)-6 being associated with worsening of cognitive function in some population studies [14–16]. However, others have not corroborated such findings [17]. Therefore, more work is needed to study the direction of this effect. As yet, the relationship between cognition and inflammation has not been studied in a CABG population.

The hormone cortisol is also posited to be a potential mechanism linking CHD and cognitive function. For example, in the cardiac literature, elevated cortisol has been linked to cardiac risk factors such as hypercholesterolemia, diabetes, abdominal obesity and hypertension by some [18, 19], but not all [20], authors. There are limited studies investigating cortisol in CABG surgery patients, although there is some evidence that there is heightened cortisol output in the post-operative period [21, 22]. Cortisol has also been linked to poorer cognitive function in observational and experimental studies [23]. Associations between the area under the curve (AUC) of cortisol and worse performance in six cognitive domains have been observed in a large cohort study [24]. Higher morning cortisol has been associated with mild cognitive impairment and a lower global cognitive state in older people [25]. In addition, a prospective association has been observed, such that high morning cortisol and a flatter slope have been associated with poorer cognitive performance 4 years later [26]. To date, only one study has investigated cognitive function and cortisol in a CABG population. Mu and colleagues [27] found that CABG patients with higher serum cortisol levels in the first post-operative morning had increased risk of cognitive dysfunction 7 days after CABG surgery. However, little is known about the impact of pre-existing cognitive state on later neuroendocrine adaptation to CABG surgery. It is possible that the neurocognitive changes resulting in impairment to memory and concentration cause systemic changes to the immune system via dysregulation of the hypothalamic-pituitary-adrenal (HPA) axis. Such changes would negatively affect the body’s ability to self-regulate following a physical trauma such as CABG surgery.

One way in which to explore the relationship between cognition and these biological mechanisms is to study the effect of pre-surgical cognitive performance on inflammatory and cortisol responses to CABG surgery. This allows the temporal relationship between cognition and biomarkers to be investigated. Therefore, the aim of the present study was to assess the prospective association between cognitive function prior to CABG surgery and subsequent inflammatory and neuroendocrine responses to surgery. Specifically, we hypothesised that poorer cognitive function would be associated with greater inflammatory responses to surgery, as measured by IL-6 and CRP, and increased cortisol output in the months following surgery, independent of mood factors and other clinical and sociodemographic confounders.

## Methods

### Participants

The study used data collected in the Adjustment and Recovery after Cardiac Surgery (ARCS) study which was designed to investigate the causes and consequences of poor emotional adjustment following cardiac surgery. Full details of the recruitment, inclusion criteria and short-term retention of participants into the ARCS study have been published elsewhere [28]. Briefly, elective, first-time candidates for CABG were recruited consecutively from a pre-surgery assessment clinic at St. George’s Hospital, London, between January 2010 and July 2012. In total, 249 participants completed valid baseline questionnaires and the Montreal Cognitive Assessment (MoCA) and gave blood samples for the measurement of IL-6 and CRP and seven saliva samples across the course of a single day for the measurement of diurnal cortisol. Participants included in these analyses were the 193 CABG surgery patients (mean age 67.46 ± 8.81 years, 9.3 % females) who provided complete data for all variables at baseline and who at follow-up provided blood for the assessment of at least one inflammatory marker and/or seven saliva samples for the measurement of cortisol. Compared to those excluded, those included in our analyses were more likely to be male (*χ*^2^ = 15.74, *p* < 0.001), but otherwise did not differ on any other sociodemographic, biomarker or clinical variable, with the minimal significance level (*p* < 0.05) used as the standard of difference. They completed baseline assessments on average 30 days prior to surgery, had blood post-operatively collected between 4 and 8 days after surgery and provided post-operative saliva samples on average 60 days after surgery.

### Measures

#### *Predictor: Cognitive Function*

The MoCA [29] was administered by the recruiting researcher in a private room at baseline. This is a brief measure of cognitive function covering the following eight cognitive domains: visuospatial awareness, executive functioning, short-term memory, attention, concentration, working memory, language and orientation to time and place. The MoCA has excellent sensitivity in identifying mild cognitive impairment (90 %) and very good specificity (87 %); it also has good test-retest reliability (*r* = 0.92, *p* = 0.001) [29]. A maximum of 31 points was awarded, with a cutoff for mild cognitive impairment of <26 [29].

#### *Outcomes: Biomarkers*

To assess inflammatory activity, we analysed blood samples collected at baseline and during the post-operative period in hospital. All participants provided a baseline sample, and the majority of participants also had blood drawn on day 4 after surgery (*N* = 111). A small number of participants (*N* = 5) had blood drawn on more than 1 day. Since there was variability in the days on which samples were collected following surgery, we created a summary score. This used the mean value for IL-6 and high sensitivity (hs)-CRP on days 4, 5, 6, 7 and 8 post-surgery. We used the mean value in order to generate a more stable aggregate score. A 20-mL blood sample was drawn into a serum separator tube by venipuncture. Post-operative bloods were collected between 6:00 and 7:00 a.m. by nursing staff. Blood was allowed to clot and centrifuged for 10 min at 3000 rpm. The resulting serum was frozen at −80 °C until batch analysis at a later date. Assays were performed by Dr David Gaze at St George’s Healthcare NHS Trust, using commercial automated immunoassay on the Immulite 1 (Siemens Healthcare Diagnostics, Frimley, Surrey). For IL-6, the minimal detectable dose was 2.0 pg/mL, the upper limit of detection was 1000.0 pg/mL and the intra-assay CV range was 3.5–6.2 %. For hs-CRP, the minimal detectable dose was 0.01 mg/dL, the upper limit of detection was 15 mg/dL and the intra-assay CV range was 4.2–6.4 %.

Saliva samples were collected using Salivettes (Sarstedt, Leicester, UK) for the assessment of cortisol. Cortisol sampling was described to participants at baseline. The collection procedure was identical at baseline and follow-up. Participants were instructed to give seven samples at set time points, on awakening, 30 min after awakening, 10:00 a.m., midday, 4:00 p.m., 8:00 p.m. and bedtime. Participants were told not to eat, drink caffeinated beverages, smoke cigarettes, take any medications or brush their teeth, for the 30 min prior to giving the sample. Participants were also required to complete a diary as a record of their sampling schedule to assess compliance. Saliva samples were obtained at baseline and 2 months after surgery and stored at −20 °C for analysis at a later date. Cortisol levels were assessed using a time-resolved immunoassay with fluorescence detection, at the University of Dresden. The intra- and inter-assay coefficients of variation were less than 4 %. Cortisol data were analysed by computing three different measures. Extreme outliers more than three standard deviations from the mean were removed. The cortisol awakening response represents the difference between the sample taken on awakening and 30 min later. To generate a valid awakening response relies on the waking sample being obtained without a delay, since otherwise the magnitude of the response will be masked [30]. Participants who reported giving their first sample more than 15 min after awakening were excluded from analyses [31]. The estimated AUC with respect to ground was generated as an approximation of total cortisol output over the day [32]. The slope of cortisol decline over the day was calculated as the reduction in cortisol per hour, using regression methods. The slope excluded the 30-min post-awakening sample, as is usual with these data [33].

#### *Covariates: Sociodemographic, Mood and Clinical Measures*

Socioeconomic status (SES) was assessed using yearly household income divided into five categories ranging from <£10,000 per year to >£40,000 per year. Education was assessed by asking participants to report their highest educational qualification attained; this was used to categorise participants into secondary education or lower and higher education and above. Pre-operative smoking was assessed, and body mass index (BMI) was measured at the pre-operative clinic appointment and calculated using the standard formula (kg/m^2^).

The Beck Depression Inventory (BDI) [34] was used to measure depression symptoms at baseline. It is a 21-item questionnaire which asks the respondent to reflect on how they have been feeling over the past 2 weeks. Ratings were summed, with higher scores indicating greater emotional disturbance, with a range of 0 to 63 (Cronbach’s *α* = 0.85).

Clinical risk was assessed using the European System for Cardiac Operative Risk Evaluation (EuroSCORE) [35]. This composite measure includes pre-surgical risk factors (e.g. age, sex and serum creatinine), cardiovascular risk factors (e.g. pulmonary hypertension and recent myocardial infarct) and operative risk factors (e.g. non-isolated CABG and surgery on the thoracic aorta). Items were scored in accordance with the ‘logistic EuroSCORE’ method to generate a percentage mortality risk estimate; further details can be found on the EuroSCORE website (www.euroscore.org/logisticEuroSCORE.htm). Cardiovascular history, clinical factors during admission and management were also obtained from clinical notes. In addition, the number of grafts a participant received and whether they underwent cardiopulmonary bypass (yes/no) were also recorded. History of diabetes was also taken from medical notes. Participants were asked to report any long-standing illnesses prior to surgery, and these were summed to capture chronic illness burden.

### Statistical Analysis

MoCA scores were treated as a binary variable (<26/≥26) since this is a clinically pertinent cutoff with high sensitivity and specificity to detect mild cognitive impairment and risk of dementia [29]. Missing data on biomarkers meant that some analyses were performed on a reduced sample size. Of all participants, baseline and post-CABG IL-6 were available for 135, hs-CRP was available for 136 and cortisol AUC was available for 131. Descriptive statistics describe the 193 participants who provided full baseline data and follow-up blood and/or saliva. Paired sample *t* tests were used to examine change in biomarkers over time. Associations between variables were assessed using Pearson’s correlations for continuous data and independent *t* tests and chi-squared tests for categorical data. Multiple linear regression was used to examine the relationship between pre-operative cognitive function and post-operative inflammatory and cortisol responses. The assumptions of regression models were assessed, including homoscedasticity, leverage points, standardised residuals and multicollinearity; all results were within the acceptable range. The independent variables in analyses were baseline scores on the MoCA, and the dependent variables were IL-6, hs-CRP and cortisol AUC. Covariates were selected on a prioi knowledge. Variables were entered into models in steps. The first model controlled for social-behavioural variables, namely education, household income, smoking status and BMI, and mood and clinical variables, namely depression, cardiopulmonary bypass use, number of grafts, chronic illness burden, diabetes and EuroSCORE. Age and sex are included in EuroSCORE, so were not entered separately to avoid double adjustment. The baseline values of each respective biomarker were also included in the first step. The second step included MoCA scores to the model. All clinical variables fell within the expected clinical range for our sample demographic [36, 37]. Follow-up IL-6 and baseline and follow-up CRP values were non-normally distributed, but since log transformation did not rectify this and since the conditions of regression were not violated, raw data were used in the analyses. Secondary analyses were performed to examine the dose–response relationship, controlling for covariates. Since the MoCA data were skewed in favour of normal cognitive function, we created a quintile variable. The quintile cutpoints were MoCA scores 23, 25, 27 and 28. Variance inflation factor values were generated for all regression models to assess multicollinearity; all were within the acceptable range. Cortisol slope (*β* = −0.065, *p* = 0.419) and awakening response (*β* = 0.095, *p* = 0.328) were not associated with cognitive function in univariate models, and therefore, the results are not reported here. All analyses were conducted using SPSS version 21. Two-tailed tests were used throughout and the significance level was set at *p* < 0.05, although exact significance levels are reported.

## Results

Table 1 summarises the characteristics of the sample. The sample had an age range between 44 and 87 years, was predominantly male (90.7 %) and overweight (BMI > 25 = 84.5 %). Participants came from a variety of income backgrounds. The majority of participants were hypertensive, and just over 20 % of participants were diabetic. The majority of participants had on-pump cardiopulmonary bypass surgery in isolation, but 43 underwent combined CABG plus valve replacement surgery. Most participants were within the normal range for depression symptoms on the BDI, but about one third (35.2 %) of participants scored >10 at baseline. Approximately 60 % of participants scored above the cutoff of 26 on the MoCA, which indicates normal cognitive functioning. Plasma IL-6 (*t* = −35.320, *p* < 0.001) and hs-CRP (*t* = −11.607, *p* < 0.001) levels increased significantly from baseline to follow-up, whilst cortisol AUC (*t* = 0.550, *p* = 0.583) responses did not change over time on average. Neither IL-6 nor hs-CRP responses were associated with cortisol at baseline or follow-up.

**Table 1:** Demographic, clinical and biological characteristics of the sample (*N* = 193)

Characteristic	Mean ± SD or *N* (%)
Age (years)	67.47 ± 8.83
Female	18 (9.4)
Ethnicity—White British/white other	172 (89.1)
Education	
Secondary or lower	143 (74.1)
Higher education or degree	50 (25.9)
BMI (kg/m^2^)	28.84 ± 4.18
Smoker	15 (7.8)
Yearly household income	
<£10,000	30 (15.6)
£10,000–19,999	56 (29.2)
£20,000–29,999	43 (22.4)
£31,000–£40,000	27 (14.1)
>£40,000	36 (18.8)
Cognitive function (MoCA) <26	77 (40.1)
BDI score	8.48 ± 6.16
Co-morbidities	
Chronic illness burden	0.44 ± 0.67
Diabetes	42 (21.9)
Hypertension	153 (79.7)
Pulmonary disease	12 (6.3)
Neurological disorder	15 (7.8)
Extracardiac arteriopathy	15 (7.8)
Clinical factors	
Logistic EuroSCORE (%)	4.19 ± 2.80
CABG in isolation	150 (78.1)
Number of grafts	3.04 ± 1.15
On-pump	148 (77.1)
Biomarkers	
Baseline IL-6 (pg/mL)*	5.23 ± 1.72
In-hospital (post-CABG) IL-6 (pg/mL)*	99.64 ± 31.45
Baseline hs-CRP (mg/dL)**	8.63 ± 9.72
In-hospital (post-CABG) hs-CRP (mg/dL)**	66.93 ± 60.87
Baseline cortisol AUC (nmol/l)***	9131.59 ± 2577.38
Two month post-CABG cortisol AUC (nmol/l)***	8955.78 ± 3532.29

*N = 135, **N = 136, ***N = 131

### Cognitive Function and Inflammatory Responses

**Fig 1:**
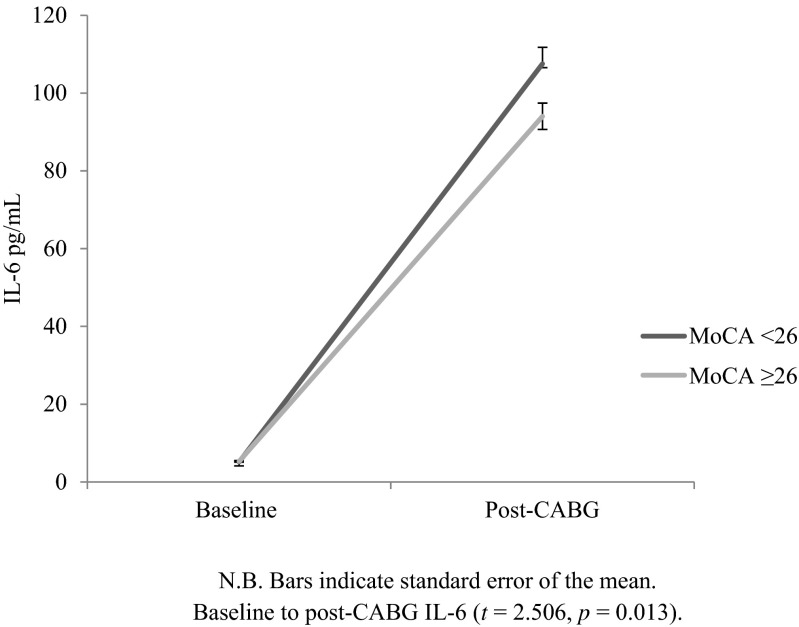
IL-6 levels (pg/mL) at baseline and follow-up by baseline MoCA score (unadjusted model)

Figure 1 shows the change in IL-6 over time by MoCA score. There was no significant difference between groups at baseline (*t* = −0.542, *p* = 0.589), but after surgery (*t* = 2.506, *p* = 0.013), there was a significant difference. Specifically, those scoring <26 on the MoCA (mean 107.54, standard deviation 31.67) had higher IL-6 concentrations than those with ≥26 MoCA scores (mean 94.04, standard deviation 30.24) in the days after CABG surgery. Table 2 displays the regression coefficients for the model predicting post-operative IL-6 responses measured after surgery. Baseline MoCA score was a significant predictor of greater IL-6 (*β* = *−*0.212, *p* = 0.021), independent of covariates. The association was negative such that pre-operative low cognitive functioning was predictive of greater IL-6 responses after surgery. The only other significant predictor in the final model was baseline IL-6 (*β* = 0.273, *p* = 0.003). The addition of MoCA to the model increased the variance explained by 3.9 %; this was a significant contribution (*p* = 0.021). The final model accounted for 14.5 % of the variance. Secondary analyses to examine the dose–response relationship between cognitive function and IL-6 showed MoCA quintile to be a significant predictor of IL-6 response after surgery (*β* = −0.198, confidence interval (CI) = −8.360–−0.216, *p* = 0.039) after controlling for covariates.

**Table 2:** Multiple regression on baseline cognitive functioning predicting post-operative IL-6 (*N* = 135)

Model	*B*	SE	*ß*	95 % CI	*P* value
Step 1					
Education	2.056	6.725	0.028	−11.256–15.369	0.760
Household income	−2.876	2.115	−0.124	−7.063–1.310	0.176
Smoking status	−3.426	9.959	−0.031	−23.140–16.287	0.731
BMI	0.677	0.653	0.093	−0.616–1.970	0.302
Baseline depression symptoms (BDI)	0.003	0.417	0.001	−0.823–0.829	0.994
Cardiopulmonary bypass	1.764	6.764	0.024	−11.625–15.154	0.795
Number of grafts	−0.659	2.539	−0.023	−5.685–4.367	0.796
Chronic illness burden	−6.894	6.077	−0.141	−18.923–5.135	0.259
Diabetes	−2.414	8.973	−0.033	−20.176–15.348	0.788
EuroSCORE	−0.129	1.060	−0.012	−2.227–1.969	0.903
Baseline IL-6	4.841	1.672	0.265	1.531–8.150	0.004
Step 2					
Education	3.131	6.622	0.042	−9.978–16.239	0.637
Household income	−1.467	2.163	−0.063	−5.748–2.814	0.499
Smoking status	−5.360	9.817	−0.049	−24.793–14.073	0.586
BMI	0.443	0.649	0.061	−0.843–1.728	0.497
Baseline depression symptoms (BDI)	−0.040	0.410	−0.009	−0.853–0.772	0.922
Cardiopulmonary bypass	2.061	6.645	0.028	−11.093–15.216	0.757
Number of grafts	−1.338	2.511	−0.048	−6.309–3.632	0.595
Chronic illness burden	−6.478	5.972	−0.133	−18.299–5.344	0.280
Diabetes	−2.212	8.814	−0.030	−19.660–15.237	0.802
EuroSCORE	−0.249	1.042	−0.022	−2.313–1.814	0.811
Baseline IL-6	4.974	1.643	0.273	1.721–8.227	0.003
Baseline MoCA score	−13.456	5.739	−0.212	−24.818–−2.094	0.021

NB step 1 *R* ^2^ = 0.107, step 2 *R* ^2^ = 0.145, ∆*R* ^2^ = 0.039, *p* = 0.021

In analyses to predict post-operative hs-CRP, baseline MoCA score (*β* = 0.064, *p* = 0.457) was not a significant predictor in basic models controlling for age and sex, and therefore, fully adjusted models were not performed.

### Cognitive Function and Cortisol AUC Responses

**Fig 2:**
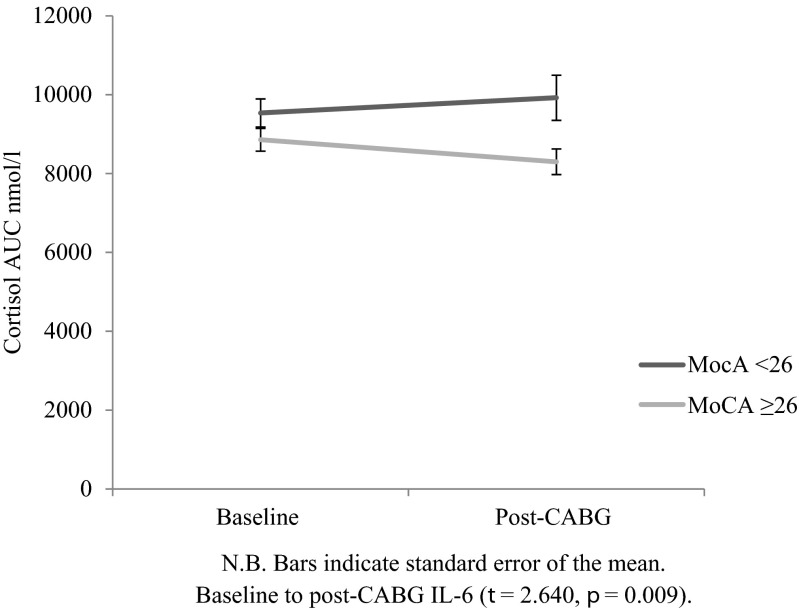
Cortisol AUC (nmol/l) at baseline and follow-up by baseline MoCA score (unadjusted model)

Figure 2 shows the change in cortisol AUC over time by MoCA score. There was no significant difference between groups at baseline (*t* = 1.487, *p* = 0.139), but differences emerged after surgery (*t* = 2.640, *p* = 0.009). Specifically, those scoring <26 on the MoCA (mean 9922.33, standard deviation 4164.34) had a higher cortisol AUC than those with ≥26 MoCA scores (mean 8299.03, standard deviation 2876.36) 2 months after CABG surgery. Table 3 displays the regression coefficients for the model predicting post-operative cortisol AUC responses measured 2 months after surgery. Baseline MoCA score was a significant predictor of greater cortisol AUC (*β* = −0.179, *p* = 0.044), independent of covariates. The association was negative such that pre-operative low cognitive functioning was predictive of greater cortisol AUC responses after surgery. The other significant predictors in the final model were smoking (*β* = 0.192, *p* = 0.023), EuroSCORE (*β* = 0.323, *p* = 0.001) and baseline cortisol AUC (*β* = 0.267, *p* = 0.003). The addition of MoCA to the model increased the variance explained by 2.6 %; this was a significant contribution (*p* = 0.044).The final model accounted for 25.5 % of the variance. Secondary analyses to examine the dose–response relationship between cognitive function and IL-6 showed MoCA quintiles to be a borderline significant predictor of cortisol AUC responses after surgery (*β* = −0.176, CI = −906.318–−0.370, *p* = 0.050) after controlling for covariates.

**Table 3:** Multiple regression on baseline cognitive functioning predicting post-operative cortisol AUC (*N* = 131)

Model	*B*	SE	*ß*	95 % CI	*P* value
Step 1					
Education	118.329	672.515	0.015	−1213.318–1449.976	0.861
Household income	174.029	242.522	0.064	−306.188–654.246	0.474
Smoking status	2582.061	1170.171	0.186	265.006–4899.116	0.029
BMI	125.660	92.776	0.123	−58.047–309.366	0.178
Baseline depression symptoms (BDI)	−58.117	52.412	−0.093	−161.899–45.665	0.270
Cardiopulmonary bypass	593.646	724.374	0.069	−840.686–2027.978	0.414
Number of grafts	−99.561	253.989	−0.033	−602.484–403.362	0.696
Chronic illness burden	150.842	670.022	0.024	−1175.868–1477.552	0.822
Diabetes	−258.176	983.143	−0.028	−2204.896–1688.545	0.793
EuroSCORE	405.255	114.325	0.338	178.880–631.630	0.001
Baseline cortisol AUC	0.401	0.119	0.293	0.166–0.637	0.001
Step 2					
Education	−114.482	673.528	−0.015	−1448.250–1219.286	0.865
Household income	338.467	252.597	0.124	−161.744–838.678	0.183
Smoking status	2667.772	1155.725	0.192	379.121–4956.423	0.023
BMI	99.524	92.464	0.097	−83.580–282.627	0.284
Baseline depression symptoms (BDI)	−56.219	51.740	−0.090	−158.678–46.239	0.279
Cardiopulmonary bypass	492.464	716.679	0.057	−926.754–1911.683	0.493
Number of grafts	−162.305	252.570	−0.054	−662.462–337.852	0.522
Chronic illness burden	195.304	661.672	0.032	−1114.986–1505.595	0.768
Diabetes	−333.939	971.075	−0.036	−2256.932–1589.053	0.732
EuroSCORE	386.407	113.217	0.323	162.206–610.608	0.001
Baseline cortisol AUC	0.366	0.119	0.267	0.131–0.601	0.003
Baseline MoCA score	−1279.758	627.825	−0.179	−2523.023–−36.494	0.044

NB step 1 *R* ^2^ = 0.229, step 2 *R* ^2^ = 0.255, ∆*R* ^2^ = 0.026, *p* = 0.044

## Discussion

The results of these analyses show that low cognitive functioning prior to surgery is predictive of impaired biological responses following CABG, including a heightened IL-6 response during the in-hospital stay and greater cortisol output up to 2 months after surgery. These results were supported in analyses using quintile MoCA scores to predict IL-6 and cortisol AUC responses, indicating a dose–response relationship. Pre-operative cognitive functioning was not associated with hs-CRP following surgery. This is the first study to our knowledge that has examined the prospective association between pre-operative cognitive functioning and biological responses to CABG surgery.

We found that approximately 40 % of participants prior to CABG surgery had low cognitive functioning as measured on the MoCA. This is in line with data from an Australian study which reported cognitive impairment in 35 % of CABG patients prior to surgery, as compared with healthy controls [5]. Variation in cardiac and demographic risk factors are likely to contribute to differences in the prevalence rates reported across different studies; however, our findings corroborate the observation that low levels of cognitive functioning are common in this patient group.

Previous studies have shown a relationship between cognitive functioning and inflammation. For example, Roberts and colleagues [13] performed a case–control study in which inflammatory markers were compared in people with and without mild cognitive impairment. These authors reported that elevated levels of plasma CRP were significantly associated with mild cognitive impairment. In our study, we were able to assess the prospective association between cognition and inflammation and found that low cognitive function promotes increased inflammation in response to CABG surgery. There is a scarcity of research investigating inflammation and cognition in cardiac patients. A longitudinal study using participants taking part in the MacArthur Studies of Successful Ageing also reported a negative association between cognitive functioning and IL-6 over time, although this was a non-clinical sample and therefore not directly comparable to our findings [15]. In our study, we only found evidence for low cognitive performance being associated with IL-6 and not CRP. The reason for this is not clear, although one possibility is that of timing. IL-6 is secreted by T cells and macrophages as part of the initial immune response and triggers the release of CRP from the liver. By measuring IL-6 and CRP at the same time, we may have captured these markers at different points in their decline towards baseline values. However, our findings warrant replication in further, large-scale studies of cardiac patients. Cognitive function explained 3.9 % of variance in IL-6 over and above other clinical, demographic and mood variables. We believe our findings to be of clinical relevance in that the heightened inflammation we observed in low cognitive function participants may in turn lead to phenomena that produce poorer recovery, such as slower wound healing or infection, or indeed, it could be important to later cardiovascular morbidity in the same way it is in acute coronary syndrome patients [38, 39]. Greater inflammation after CABG has previously been associated with greater morbidity including increased risk of atrial fibrillation [40], poorer lung function [41] and even death [42]. This hypothesis requires further testing using other longer-term CABG morbidity end points. We have shown in other analyses that the magnitude of acute inflammatory responses following surgery predicts later depressive symptoms [43] and length of hospital stay [44].

Previous research has shown that cognitive function, particularly memory, and cortisol levels are related [45–47]. Whilst this literature is well established, few studies have investigated these effects in CABG patients. One study examined 166 patients after CABG surgery and found that higher levels of serum cortisol on the morning after surgery was associated with poorer cognitive performance 7 days later [27]. Salivary cortisol has been found to increase greatly after non-cardiac surgery in association with post-operative cognitive dysfunction [48], so this effect might not be specific to CABG surgery patients. In our study, we found that low pre-operative cognitive function predicts greater cortisol output across the day up to 2 months after CABG surgery. Indeed, cognitive function predicted 2.6 % of variance in cortisol AUC responses over and above covariates. There is limited evidence for the detrimental effects of elevated cortisol in this patient group; however, there is some evidence that it is associated with poorer lung function [41] and increased risk of delirium [49]. Moreover, we recently showed that flatter cortisol slopes prior to CABG were associated with greater mortality and major adverse events up to 2.68 years after surgery [50]. We found an effect for cortisol AUC but not for cortisol awakening response or slope. The reason for this is not entirely clear, although previous work has suggested that the awakening response in particular is highly influenced by situational factors [51], making it a less reliable indicator of trait responses. Further research is needed to understand whether these effects persist in the longer term and whether such elevations in cortisol translate into greater clinical risk in these patients.

Interestingly, we showed that not only those participants falling below the cutoff for mild cognitive impairment were at risk of poorer biological responses but also that cognition and inflammation and cortisol were associated in a graded fashion, indicative of a dose–response relationship. This suggests that even amongst participants within the normal range of cognitive performance, those scoring at the lower end of the scale had worse biological responses than those at the higher end of the scale. More work is needed to understand the use of cutoffs in this patient group and the benefits of early identification of patients with less than optimal cognitive functioning.

In the current study, heightened inflammatory and neuroendocrine activity after the stress of CABG surgery seen in patients with lower cognitive functioning suggest that there may be disruption of the HPA axis in some patients. The human stress response involves activation of the HPA axis, the subsequent release of cortisol and simultaneous elevation of inflammatory cytokines [52]. However, we were unable to support the association between cortisol and inflammation using our data, perhaps due to the temporal differences in collection of blood and saliva samples. Further research examining these biomarkers in CABG patients is needed. In addition, non-biological mechanisms also warrant further examination since behavioural pathways including the role of physical activity, adherence to medications, diet and alcohol consumption may also be relevant. Psychological mechanisms are also likely to be important. It is possible that those with cognitive impairment are less likely to employ adaptive coping responses (e.g. social support) to a stressor such as CABG surgery, resulting in greater perceived stress and in turn poorer biological responses to surgery. These pathways require further study as potential mediators or moderators of this relationship.

There are several strengths to our study. The longitudinal design of the ARCS study allows for the temporal relationship between cognitive function and biological responses to surgery to be analysed. Moreover, the large range of psychosocial and clinical data collected from the ARCS participants has allowed us to control for multiple covariates that might confound the association between cognition and biomarkers. However, there are also a number of limitations. Firstly, we have relied on a short, global assessment of cognitive function that does not allow us to study the relationship between different cognitive domains and inflammation and cortisol. In addition, cognitive function was only assessed pre-operatively, so it was not possible to assess the effect of cognitive deterioration from before to after the surgical procedure. Moreover, missing data on the biological measures meant that we had to perform some of our analyses on a reduced sample size. The ARCS study had a relatively homogenous sample, being predominately male and of White ethnic origin making generalisation to other groups difficult. Only 9.4 % of our samples were female. This male majority is characteristic of the CABG surgical population more generally, with men more likely to receive a revascularisation procedure than women in the UK. However, we included sex in all analyses as a covariate in order to address this issue.

In conclusion, we found that low cognitive functioning prior to CABG surgery was associated with greater IL-6 responses in the days following surgery and greater cortisol output up to 2 months later. The poorer biological responses observed in these patients may help explain the link between cognitive decline and cardiovascular pathology.
